# Clip-Domain Serine Protease Gene (*Ls*CLIP3) Is Essential for Larval–Pupal Molting and Immunity in *Lasioderma serricorne*

**DOI:** 10.3389/fphys.2019.01631

**Published:** 2020-01-31

**Authors:** Wen-Jia Yang, Chun-Xu Chen, Yi Yan, Kang-Kang Xu, Can Li

**Affiliations:** Guizhou Provincial Key Laboratory for Rare Animal and Economic Insect of the Mountainous Region, College of Biology and Environmental Engineering, Guiyang University, Guiyang, China

**Keywords:** clip-domain serine protease, *Lasioderma serricorne*, RNA interference, molting, immunity

## Abstract

Clip-domain serine proteases (CLIPs) play crucial roles in insect development and innate immunity. In this study, we identified a CLIP gene (designated *Ls*CLIP3) from the cigarette beetle *Lasioderma serricorne*. *Ls*CLIP3 contains a 1,773-bp open reading frame (ORF) encoding a 390-amino-acid protein and shows a conserved clip domain and a trypsin-like serine protease domain. Phylogenetic analysis indicated that *Ls*CLIP3 was orthologous to the CLIP-B subfamily. *Ls*CLIP3 was prominently expressed in larva, pupa, and early adult stages. In larval tissues, it was highly expressed in the integument and fat body. The expression of *Ls*CLIP3 was induced by 20-hydroxyecdysone. A similar induction was also found by peptidoglycans from *Escherichia coli* and *Staphylococcus aureus*. RNA interference (RNAi)-mediated silencing of *Ls*CLIP3 disrupted larval–pupal molting and specifically reduced the expression of genes in 20-hydroxyecdysone synthesis and signaling pathway. The chitin amounts of *Ls*CLIP3 RNAi larvae were greatly decreased, and expressions of six chitin metabolic-related genes were significantly reduced. Knockdown of *Ls*CLIP3 increased larval sensitivity to Gram-negative and Gram-positive bacteria. There was significantly decreased expression of four antimicrobial peptide (AMP) genes. The results suggest that *Ls*CLIP3 is an important component of the larva to pupa molt and for the immunity of *L. serricorne*.

## Introduction

Serine proteases (SPs) comprise a large family of proteolytic enzymes. They are characterized by a conserved His–Asp–Ser catalytic triad active site domain. SPs participate in physiological processes such as digestion, immune responses, fertilization, and embryonic development (Lu et al., 2014; Veillard et al., 2016). SP homologs (SPHs) are a family of enzymes with a similar amino acid sequence as SPs but lacking the amidase activity required for the substitution of catalytic residues (Ross et al., 2003). A well-characterized chymotrypsin-like peptidase, SPH plays a critical role in the molting of *Tribolium castaneum* (Broehan et al., 2010). SPs and SPHs constitute a large family of proteases in insects, with 50−300 genes in genomes (Veillard et al., 2016). The clip-domain SPs (CLIPs) are the most important multiple-domain SPs. They are extracellular and characterized by one or more clip domain(s) at the amino terminus. In each clip domain, 37−55 amino acid residues, including six strictly conserved cysteine residues, are arranged in three disulfide-bridge structures (Jiang and Kanost, 2000). The CLIP was first identified in the hemolymph pathway of the horseshoe crab *Tachypleus tridentatus* (Muta et al., 1990). CLIPs have now been identified and characterized in many insects. CLIPs, and occasionally clip-domain SPHs, act in the final steps of SP pathways in insects (Jiang and Kanost, 2000).

Many CLIPs have been identified in many insect species at the genome level. For example, 42 CLIPs (including clip-domain SPHs) are known in *Drosophila melanogaster* (Ross et al., 2003; Veillard et al., 2016), 18 in *Bombyx mori* (Tanaka et al., 2008; Zhao et al., 2010), 42 in *Manduca sexta* (Zou et al., 2007; Cao et al., 2015), 13 in *Plutella xylostella* (Lin et al., 2015), and 25 in *Pteromalus puparum* (Yang et al., 2017). CLIPs represent a large gene family in insects, which can be divided into four distinct clades. These enzymes are named CLIP-A, CLIP-B, CLIP-C, and CLIP-D based on the number of residues and predicted secondary structure between Cys3 and Cys4 (Jiang and Kanost, 2000; Ross et al., 2003). Each clade represents a multigene family (Kanost and Jiang, 2015). CLIPs are non-digestive enzymes present in the hemolymph and acting as extracellular signal components and mainly function in insect development and innate immunity (Jiang and Kanost, 2000; Kanost and Jiang, 2015; Veillard et al., 2016). In *Drosophila*, CLIPs are involved in the establishment of the dorsal−ventral axis during embryogenesis (Morisato and Anderson, 1995). Microbial infection can activate CLIP activity and activate prophenoloxidase. This is involved in melanization and also the activation of the Toll signal that produces downstream antimicrobial peptides (AMPs) (Kanost and Jiang, 2015). In *P. puparum*, 5 of 25 CLIPs were upregulated by pathogen infection (Yang et al., 2017). Other roles of CLIPs in insects have been reviewed (Veillard et al., 2016). In the molting fluid of *B. mori*, four CLIPs were identified using proteomics (Zhang et al., 2014). Two CLIPs, CLIP8 and CLIP11, had an evolutionary relationship with homologs that were involved in the immune response (Kanost et al., 2001). The *BmSP95* CLIP is involved in the pupa to adult molt (Liu et al., 2017). While this is a large family of enzymes, limited functions have been documented, and the roles of most CLIPs remain unknown.

The cigarette beetle, *Lasioderma serricorne* (Fabricius), is an important pest of stored products worldwide. It destroys stored tobacco, grains, spices, and other agricultural products (Mahroof and Phillips, 2008). The adults are short lived, but lay eggs in stored products. Larvae feed on stored products and reduce, or destroy, product quality. Little is known about the immunity and molting-related genes in *L. serricorne*. In a previous study, we identified two *LsCLIPs* in *L. serricorne* (Chen et al., 2019) and demonstrated their transcriptional responses to 20-hydroxyecdysone (20E) and pathogen infection. In this study, the full length of a *CLIP* gene (*Ls*CLIP3) was cloned in *L. serricorne*. The expression profiles of *Ls*CLIP3 in different developmental stages and tissues were detected by quantitative real-time PCR (qPCR). Expression responses of *Ls*CLIP3 to 20E exposure and immune challenges were also examined. Furthermore, RNA interference (RNAi) was used to clarify the role of *Ls*CLIP3 in *L. serricorne* larval development and tolerance to immune triggers.

## Materials and Methods

### Insect Culture

The laboratory strain of *L. serricorne* was originally obtained from a tobacco warehouse in Guizhou Province, China. The beetles were reared on *Angelica sinensis*, at 28°C with 40% relative humidity and constant 24 h dark (0L:24D) conditions as described previously (Cao et al., 2019; Yang et al., 2019).

### Complementary DNA Cloning of *Ls*CLIP3

Total RNA was extracted from *L. serricorne* adults using the MiniBEST Universal Extraction Kit (TaKaRa, Dalian, China) and treated in a gDNA Eraser spin column to remove genomic DNA. RNA was quantified with a NanoDrop 2000C spectrophotometer (Thermo Fisher Scientific, Waltham, MA, United States), and the integrity was evaluated by agarose gel electrophoresis. First-strand complementary DNA (cDNA) was synthesized using TransScript Synthesis Supermix (TransGen, Beijing, China) following manufacturer’s instructions. The fragment of clip-domain serine protease (CLIP) gene was identified from the transcriptome of *L. serricorne* and further analyzed by the online ORF Finder^1^. Specific primers (Supplementary Table 1) were designed to amplify the full length of ORF amplification. The PCR conditions were 95°C for 5 min, 35 cycles of 95°C for 30 s, 58°C for 30 s, and 72°C for 2 min, and then an extension of 72°C for 10 min. After the purification, the PCR products were transformed into *Escherichia coli* Tans1-T1 cells and sequenced by Tsingke Biotechnology Company (Chengdu, China).

### Sequence Analysis

Sequence similarities were ascertained with the BLAST program^2^. The molecular weight and isoelectric point were calculated using ExPASy tools^3^. The potential *Ls*CLIP3 protein sequence were further predicted for the presence of conserved domains using the Simple Modular Architecture Research Tool^4^ and for signal peptide prediction using SignalP5.0 Server^5^. Multiple sequence alignment was performed using DNAMAN7.0 (Lynnon Biosoft, QC, Canada). The phylogenetic tree was constructed using MEGA7.0 by the neighbor-joining method using bootstrap analysis with 1,000 replications (Kumar et al., 2016).

### Developmental and Tissue-Specific Expression of *Ls*CLIP3

Samples were prepared to study the temporal and spatial expression pattern of *Ls*CLIP3 in *L. serricorne*, as previously reported (Chen et al., 2019). The samples from developmental stages included early instar larvae, late instar larvae, pupae, early adults, and late adults. Each sample contained 30–40 individuals and repeated three times. For the tissue-specific experiment, four tissues (integument, midgut, fat body, and Malpighian tubules) were dissected from the fifth instar larvae of *L. serricorne*. Forty larvae were collected for tissue dissection in one replicate, and three biological replications were applied. The methods of RNA extraction and cDNA synthesis were the same as above. The qPCR was carried out on a CFX-96 real-time detection system (Bio-Rad, Hercules, CA, United States) in 20 μl reaction containing 10 μl of GoTaq^®^ qPCR Master Mix (Promega), 1 μl of cDNA template, 1 μl of each gene-specific primers, and 7 μl nuclease-free water. The thermocycling program was as follows: 95°C for 5 min followed by 40 cycles of 95°C for 5 s and 60°C for 34 s. A melting curve analysis was performed for all reactions from 60 to 95°C to ensure the specificity of the primers. The *L. serricorne 18S rRNA* gene (*18S*, GenBank: MK033476) was amplified for internal standardization. The relative expression levels of *Ls*CLIP3 were calculated by the 2^–ΔΔ*Ct*^ method (Livak and Schmittgen, 2001).

### Expression of *Ls*CLIP3 Induced by 20-Hydroxyecdysone and Immune Triggers

A sample of 20E (Sigma-Aldrich, St Louis, MO, United States) was dissolved in distilled water with added 95% ethanol to produce a stock solution of 10 μg/μl, and further diluted with distilled water to a working solution of 1 μg/μl. Each late larva was injected with 120 nl 20E (1 μg/μl). Control larvae were injected with an equal volume of 0.1% ethanol. Treated larvae were collected at 4, 8, and 12 h after injection. For immune challenge experiments, two peptidoglycans, PGN-SA and PGN-EB (InvivoGen, San Diego, CA) from the Gram-positive bacterium *Staphylococcus aureus* and Gram-negative bacterium *E. coli*, were diluted in sterilized endotoxin-free water to a final working solution (1 μg/μl). Approximately 200 nl of PGN-SA solution or PGN-EB solution was injected into the body cavity of fourth instar larva as described previously (Chen et al., 2019). The control larvae were injected with 200 nl sterile endotoxin-free water. Thirty individuals were randomly sampled at 3, 6, and 9 h after injection. All of the above samples were collected for total RNA isolation and gene expression determination by qPCR. Each time point was repeated three times.

### Synthesis of Double-Stranded RNA Targeting *Ls*CLIP3 and Injection of dsRNA

RNA interference was performed to study the potential function of *Ls*CLIP3 in *L. serricorne*. Double-stranded RNAs (dsRNAs) of *Ls*CLIP3 and green fluorescent protein (*GFP*, as control) were prepared *in vitro* using TranscriptAid T7 High Yield Transcription Kit (Thermo Fisher Scientific, United States). Specific primers used for dsRNA synthesis are listed in Supplementary Table 1. Approximately 300 ng of ds*Ls*CLIP3 or ds*GFP* was slowly injected into the hemocoel between the second and third abdominal segments of each fourth instar larva using a Nanoliter 2010 injector (World Precision Instruments, Sarasota, FL, United States). Three biological replicates (60 individuals each) were treated by ds*Ls*CLIP3 and ds*GFP* injection. The insects treated with dsRNA were reared under standard conditions.

### Effect of *Ls*CLIP3 RNAi on the Transcripts of Ecdysone Synthesis and Signaling Genes

Tested insects were collected at 3 and 5 days after injection, and total RNAs were extracted for RNAi efficiency determination. Insect survival rate and phenotype changes were recorded. The phenotypes were captured using a Keyence VHX-6000 stereomicroscope (Keyence Corporation, Osaka, Japan). After *Ls*CLIP3 was knocked down, transcript levels of four ecdysone synthesis-related genes (*LsCYP302a1*, *LsCYP306a1*, *LsCYP314a1*, and *LsCYP315a1*) and six ecdysone signaling genes, including *ultraspiracle* (*LsUSP*), *ecdysone-induced protein 74* (*LsE74*), *ecdysone-induced protein 75* (*LsE75*), *Krüppel-homolog 1* (*LsKr-h1*), *hormone receptor 38* (*LsHR38*), and ftz transcription factor (*LsFTZ-F1*) were detected by qPCR at 5 days after injection.

### Effects of *Ls*CLIP3 RNAi on Chitin Content and Transcripts of Chitin Metabolic Genes

To determine the effect of *Ls*CLIP3 depletion on the chitin metabolism, we collected whole insect bodies at 5 days after injection to assay the chitin content (Xia et al., 2016). Briefly, samples were mixed with 0.5 g zirconium beads (BioSpec Products, Bartlesville, OK, United States) and 0.3 ml KOH (14 M) and were then homogenized. The homogenates were heated at 130°C for 1 h, followed by centrifugation at 12,000 × *g* for 20 min and discarding the supernatant. The pellet was suspended in 3% sodium dodecyl sulfate and centrifuged for 10 min at 12,000 × *g*, and supernatant was discarded. The pellet was individually mixed with 10% KHSO_4_ and NaNO_2_ and incubated at 25°C for 15 min to detect the glucosamine residues and depolymerize the chitosan. After treatment with 12.5% NH_4_SO_3_NH_2_ (Sigma-Aldrich, United States) and 3-methyl-1-2-benzothiazolone hydrazone hydrochloride hydrate (MBTH, 5 g/L), 0.83% FeCl_3_ was added as chromogenic agent. Chitin content was measured by a SpectraMax M2 microplate reader (Molecular Devices, Sunnyvale, CA, United States) and calculated according to the established standard curve concentrations of glucosamine (Sigma-Aldrich). The results were given as units of chitin content (microgram) per milligram fresh body. To determine the expression of chitin metabolic genes, samples were collected from the larvae injected with ds*Ls*CLIP3 and ds*GFP* for 5 days. The relative expression levels of five chitin synthesis genes, including *trehalase* (*LsTRE1* and *LsTRE2*), *UDP-N-acetylglucosamine pyrophosphorylase* (*LsUAP1* and *LsUAP2*), and *chitin synthase 1* (*LsCHS1*), and four chitin degrading genes, *chitin deacetylase 1* (*LsCDA1*), β*-N-acetylglucosaminidase* (*LsNAG1* and *LsNAG2*), and *chitinase 5* (*LsCHT5*), were determined by qPCR.

### Transcripts of Immune-Related Genes and Bioassays With Bacteria After RNAi

To evaluate the role of *Ls*CLIP3 in immune defense, the expression profiles of six immune-related genes, including *lysozyme* (*LsLys1* and *LsLys2*), *coleoptericin* (*LsCol*), *defensin* (*LsDef1* and *LsDef2*), and *attacin* (*LsAtt*) were detected by qPCR. The *Ls*CLIP3 suppressed beetles were collected and injected with living Gram-negative bacteria (*E. coli* DH5α) and Gram-positive bacteria (*S. aureus*) after 72 h injection of dsRNA. Each larva was injected with 200 nl of *E. coli* solution (OD_600_ = 2.0) or *S. aureus* (OD_600_ = 1.0). The same volume of sterile endotoxin-free water was injected in the negative control insects. The mortality rates of RNAi insects, after bacteria challenge, was recorded 48 h after treatment. Fifty insects were injected as one replication, and three replications were performed in each treatment.

### Statistical Analysis

All data were analyzed using the SPSS 20.0 software (IBM Corp., Chicago, IL, United States) and presented as mean ± standard error. Differences in gene expression levels were analyzed using one-way analysis of variance (ANOVA) followed by a least significant difference test. The difference in gene expression between 20E treatment, bacteria treatment, and silencing efficiency compared with controls were determined using the Student’s *t* test. Differences were considered as significant at a *P* < 0.05.

## Results

### Identification and Characterization of *Ls*CLIP3

**FIGURE 1:**
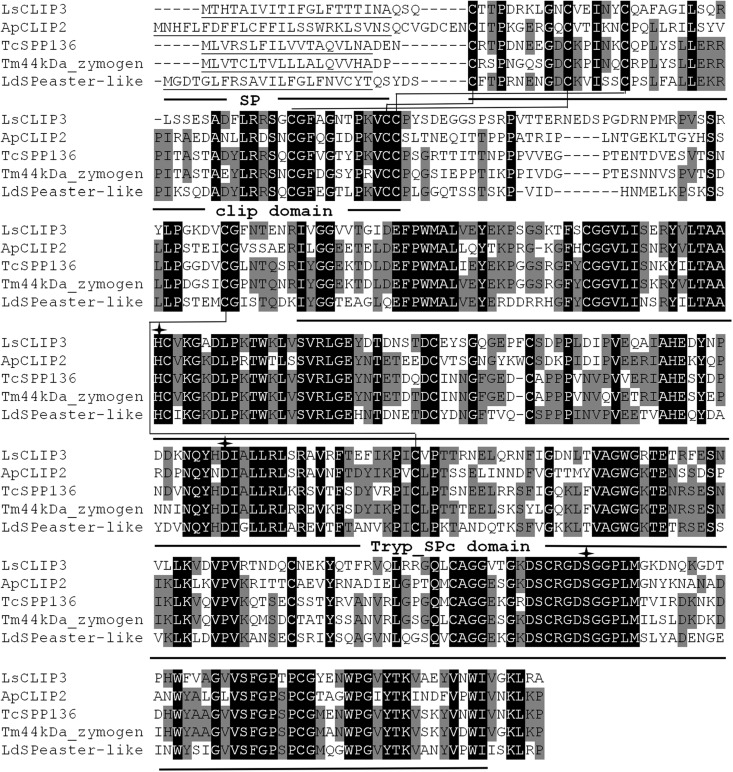
Multiple alignments of the amino acid sequence of *Ls*CLIP3 with homologs from other insect species. Black represents 100% identity, and gray represents ≥60% identity. The predicted signal peptides (SP), Clip domains, and trypsin-like serine protease (Tryp-SPc) domains are underlined. The conserved disulfide bonds are in bold and labeled with connecting lines. The amino acids of the catalytic triad are marked with arrows. ApCLIP2 (*Agrilus planipennis*; GenBank accession number: XP_018332507.1), TcSPP136 (*Tribolium castaneum*, EFA07558.1), Tm44kDa_zymogen (*Tenebrio molitor*; BAG14262.2), and LdSPeaster-like (*Leptinotarsa decemlineata*; XP_023025567.1).

The full ORF of the CLIP (designated as *Ls*CLIP3, GenBank accession number MK015721) sequence was confirmed by reverse transcription PCR. *Ls*CLIP3 has a 1,173-bp ORF encoding 390 amino acid residues with a calculated molecular weight of 43.0 kDa and a theoretical isoelectric point of 5.85. A signal peptide with 20 amino acids were found at the N-terminal ends of LsCLIP3. The predicted amino acid sequence of *Ls*CLIP3 contained a typical clip domain and a catalytic domain. Six cysteine residues forming three disulfide linkages of clip domain were at the N-terminus, and the catalytic His–Asp–Ser triad was found in *Ls*CLIP3. The conserved trypsin-like serine protease domain (Tryp_SPc domain) at the C-terminal region was also identified (Figure 1).

**FIGURE 2:**
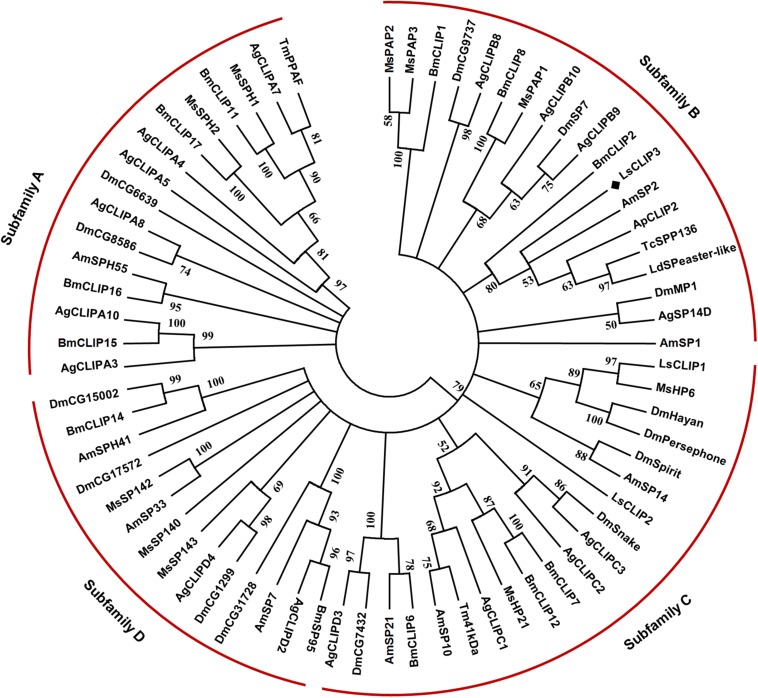
Phylogenetic analysis of LsCLIP3 and its homologs in insects. MEGA 7.0 was used to construct the phylogenetic tree by the neighbor-joining method. Bootstrap analysis was performed with 1,000 replicates. Ap, *Agrilus planipennis*; Ag, *Anopheles gambiae*; Am, *Apis mellifera*; Bm, *Bombyx mori*; Dm, *Drosophila melanogaster*; Ls, *Lasioderma serricorne*; Ld, *Leptinotarsa decemlineata*; Tc, *Tribolium castaneum*; Tm, *Tenebrio molitor*. The black diamond stands for protein sequence of LsCLIP3 from *L. serricorne*. The sequence presented in the phylogenetic tree can be found in Supplementary Table 2.

Sequence alignment showed that *Ls*CLIP3 shared 58.0, 56.3, 52.5, and 51.9% identity with the serine proteases of *Tenebrio molitor* (BAG14262.2), *T. castaneum* (EFA07558.1), *Leptinotarsa decemlineata* (XP_023025567.1), and *Agrilus planipennis* (XP_018332507.1), respectively. A phylogenetic tree was constructed with 69 CLIPs from other insects. These proteins were grouped into four subfamilies (CLIP-A, CLIP-B, CLIP-C, and CLIP-D). *Ls*CLIP3 formed a cluster with BmCLIP2 from *B. mori*, AmSP2 from *Apis mellifera*, ApCLIP2 from *A*. *planipennis*, TcSPP136 from *T. castanum*, and LdSPeaster-like from *L*. *decemlineata*, and they were grouped into the CLIP-B subfamily (Figure 2).

### Developmental and Tissue-Specific Expression of *Ls*CLIP3

**FIGURE 3:**
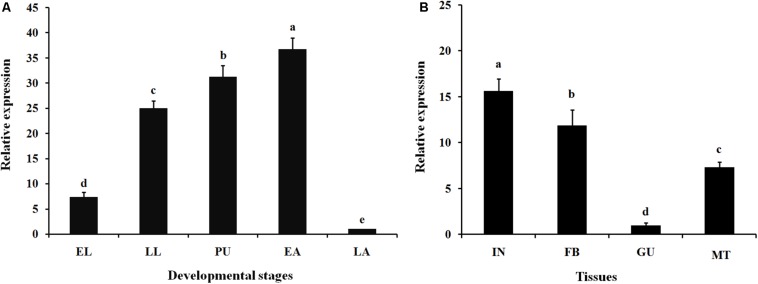
Relative expression of *Ls*CLIP3 in different developmental stages **(A**) and different larval tissues **(B)** of *Lasioderma serricorne*. EL, LL, PU, EA, and LA represent early instar larvae, late instar larvae, pupae, early adults, and late adults, respectively. IN, FB, GU, and MT represent integument, fat body, gut, and Malpighian tubules, respectively. Different letters above bars indicate significant differences based on one-way ANOVA followed by a least significance difference test (*P* < 0.05).

Expression profiles of *Ls*CLIP3 in the developmental stages and tissues of *L. serricorne* were detected by qPCR. *Ls*CLIP3 was consistently expressed in all test stages but was most highly expressed in early adults, pupae, and late instar larvae (Figure 3A). *Ls*CLIP3 was moderately expressed in early instar larvae and least expressed in late adults. Among the tissues of the fifth instar larvae, *Ls*CLIP3 was predominantly found in the integument, but high levels also occurred in the fat body. There was low expression in other tissues (Figure 3B).

### Expression of *Ls*CLIP3 in Response to 20E and Peptidoglycans Induction

**FIGURE 4:**
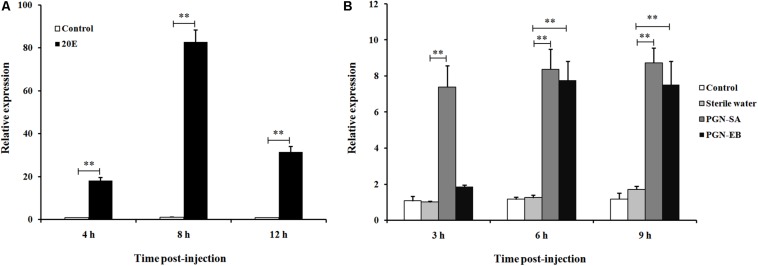
Relative expression of *Ls*CLIP3 in *Lasioderma serricorne* larvae after being induced by 20E **(A)** and peptidoglycans **(B)**. PGN-SA and PGN-EB represents peptidoglycan from the Gram-positive bacterium *Staphylococcus aureus* and Gram-negative bacterium *Escherichia coli*, respectively. The black control larvae were pricked with microinjection needle but not subjected to injection, and the sterile water group was negative control. Significant differences between the treatment group and the negative control group at the same point were determined using Student’s *t* test (^∗∗^*P* < 0.01).

To determine whether *Ls*CLIP3 is induced by 20E, the late larvae were treated with 20E for 4, 8, and 12 h. The results showed that the expression of *Ls*CLIP3 was significantly increased after treatment with 20E compared to that of control in three time points. The expression of *Ls*CLIP3 increased gradually from 4 h, peaking at 8 h, and then declined slightly at 12 h (Figure 4A).

The expression of *Ls*CLIP3 in the PGN-SA challenged group was significantly increased, and the ratios of inducible expression were 7.3-, 8.2-, and 8.8-fold at 3, 6, and 9 h post-injection, respectively, compared to the water control group (Figure 4B). The expression of *Ls*CLIP3 was significantly increased up to 7.7- and 7.2-fold at 6 and 9 h after PGN-EB injection compared to the water control. However, *Ls*CLIP3 showed no significant increase at 3 h after PGN-EB treatment (Figure 4B). There were no differences between the negative control water-treated group and the blank control group.

### Knockdown of *Ls*CLIP3 Impairs Larval–Pupal Molting

**FIGURE 5:**
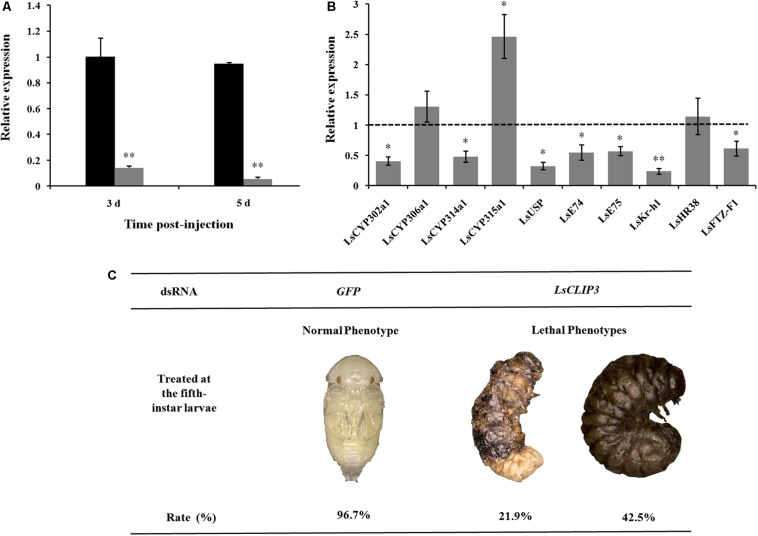
Effect of *Ls*CLIP3 knockdown on the larval–pupal molting in *Lasioderma serricorne*. RNAi efficiency of *Ls*CLIP3 by dsRNA injection into the larva **(A)**. Effects of *Ls*CLIP3 knockdown on the expression of genes involved in 20E biosynthesis and signaling pathways **(B)**. The phenotype of the larva after dsRNA injection **(C)**. The expression values of 20E synthesis and signaling pathway genes were calculated by comparison to the control, which was normalized at 1. Significant differences were identified by Student’s *t* test (^∗^*P* < 0.05, ^∗∗^*P* < 0.01).

After dsRNA delivery, *Ls*CLIP3 expression levels were significantly reduced by 87 and 92% at 3 and 5 days, respectively (Figure 5A). We further detected the expression of four ecdysone synthesis genes and six ecdysone signaling pathway genes in larvae using qPCR after *Ls*CLIP3 silencing. At 5 days after injection with ds*Ls*CLIP3, the mRNA levels of *LsCYP302a1* and *LsCYP314a1* were significantly decreased (Figure 5B). The expression of ecdysone-inducible genes, including *LsUSP*, *LsE74*, *LsE75*, *LsKr-h1*, and *LsFTZ-F1*, were significantly decreased. However, ds*Ls*CLIP3 had no apparent influence on the transcription level of *LsHR38*. The survival rate of *L. serricorne* was reduced to 35.6% in ds*Ls*CLIP3 group, whereas 96.7% of the larvae in the control group (ds*GFP*-injected larvae) were alive and molted normally to pupae (Figure 5C). Knockdown of *Ls*CLIP3 resulted in 21.9% larvae that were unable to molt successfully. These larvae retained their larval form, and the cuticle was crimped with a twisted body; 42.5% of the individuals turned black and died with abnormal pigmentation (Figure 5C).

### Knockdown of *Ls*CLIP3 Affects Chitin Accumulation

**FIGURE 6:**
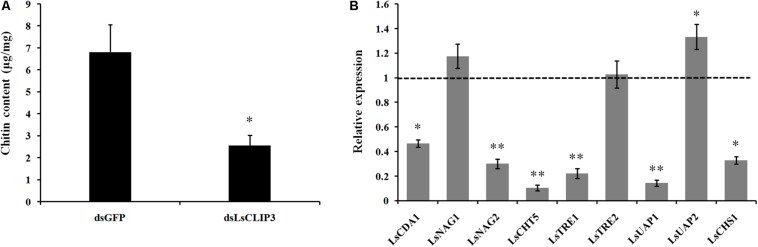
Effect of *Ls*CLIP3 knockdown on chitin content **(A)** and expression of nine genes involved in chitin metabolism **(B)**. Significant differences were identified by Student’s *t* test (^∗^*P* < 0.05, ^∗∗^*P* < 0.01).

We also used RNAi to investigate the role of *Ls*CLIP3 in chitin metabolism of *L. serricorne*. Chitin contents were reduced in the *Ls*CLIP3 RNAi larvae compared to the controls (Figure 6A). Consistently, the mRNA levels of three chitin degrading genes (*LsCDA1*, *LsNAG2*, and *LsCHT5*) and three chitin synthesis genes (*LsTRE1*, *LsUAP1*, and *LsCHS1*) were significantly reduced in *Ls*CLIP3-depleted larvae (Figure 6B). These data suggest that *Ls*CLIP3 is involved in the regulation of genes that take part in chitin synthesis and degradation during larva to pupa molt.

### Effects of *Ls*CLIP3 RNAi on Sensitivity Against Bacteria Infection

**FIGURE 7:**
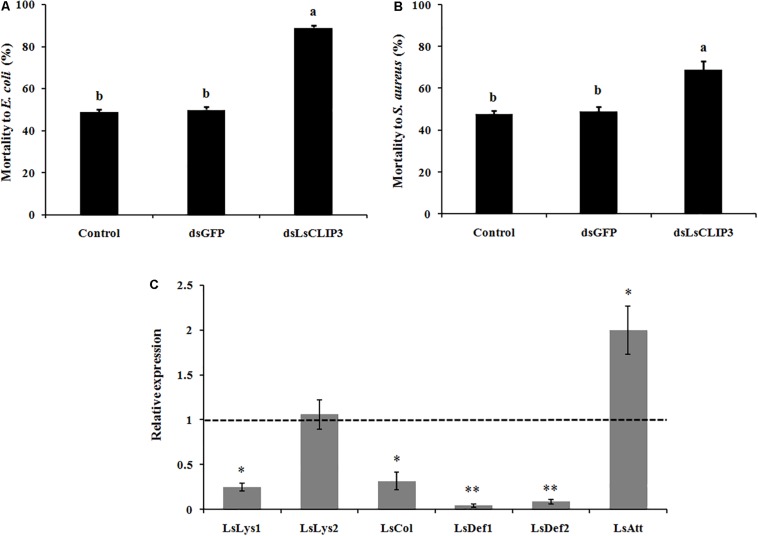
Effect of *Ls*CLIP3 knockdown on sensitivity against Gram-negative **(A)** and Gram-positive bacteria **(B)** infection, and the expression of six antimicrobial peptide genes **(C)**. Different letters above bars indicate significant differences among the mortalities based on one-way ANOVA followed by a least significance difference test (*P* < 0.05). Significant differences between the RNAi group and the control group were identified by Student’s *t* test (^∗^*P* < 0.05, ^∗∗^*P* < 0.01).

The role of *Ls*CLIP3 in resistance against bacteria pathogens was determined by measuring mortality rates after RNAi of *Ls*CLIP3 in *L. serricorne* larvae. Knockdown of *Ls*CLIP3 increased the mortality of *L. serricorne* larvae exposed to Gram-negative *E. coli* and Gram-positive *S. aureus* (Figures 7A,B). After exposure to *E. coli* and *S. aureus*, at the LC_50_ doses, larvae injected with ds*Ls*CLIP3 (compared to ds*GFP*) significantly increased larval mortality rate caused by *E. coli* (from 48.9 to 88.9%), or *S. aureus* (from 47.8 to 68.9%). Expression levels of four AMP genes (*LsLys1*, *LsCol*, *LsDef1*, and *LsDef2*) greatly declined in the *Ls*CLIP3 RNAi larvae compared with those in the controls (Figure 7C).

## Discussion

Extracellular SP cascades have evolved in invertebrates to mediate physiological or pathological cues. The CLIPs contain one or multiple conserved Clip domains. It has been demonstrated that CLIPs play important roles in insect development and innate immune responses (melanizatin and AMP production) (Kanost and Jiang, 2015). CLIPs are non-digestive serine proteases present in insect hemolymph, which were identified and characterized in *Anopheles gambiae* (Christophides et al., 2002), *Aedes aegypti* (Waterhouse et al., 2007), *B. mori* (Zhao et al., 2010), *P. puparum* (Yang et al., 2017), and *M. sexta* (Cao et al., 2015). The structure and functions of CLIPs in *Drosophila* were reviewed recently (Veillard et al., 2016).

### Gene Characterization of *Ls*CLIP3 in *L. serricorne*

We previously identified two CLIPs in *L. serricorne* (*LsCLIP1* and *LsCLIP2*) belonging to the CLIP-C subfamily (Chen et al., 2019). Here, we report a new CLIP gene (*Ls*CLIP3) in *L. serricorne*, which contain a conserved Clip catalytic domain. Multiple sequence alignment revealed high similarity of LsCLIP3 to CLIPs from other Coleoptera such as *T. moliter* and *T. castaneum*. Since insect CLIPs are extracellular serine proteases, LsCLIP3 protein could be likely a prefixed signal peptide. Similar to the *Octodonta nipae* CLIPs (Zhang et al., 2017), the trypsin-like serine protease domain at the carboxyl-terminus was also characterized in the deduced protein of *Ls*CLIP3. The CLIPs in arthropods are placed into four subfamilies, CLIP-A, CLIP-B, CLIP-C, and CLIP-D (Cao et al., 2015). In insects, the CLIPs with known functions occur in the CLIP-B and CLIP-C subfamilies, based on the overall sequence similarity and structural features of the Clip domains (Kanost and Jiang, 2015). Phylogenetic analysis indicates that LsCLIP3 belongs to the CLIP-B subfamily clade, which differs from the previous two *LsCLIPs* assigned to the CLIP-C subfamily (Chen et al., 2019). Genes in the CLIP-B and CLIP-C subfamilies can be distinguished based on their position in the cascade pathway (An et al., 2009). In addition, the length between the third and fourth Cys is different so that it can be used to define the two subfamilies (Jiang and Kanost, 2000). In *L. serricorne*, there are 15−17 amino acids in LsCLIP1 and LsCLIP2, while there are 24 in LsCLIP3 protein. This is consistent with the definition of the type of the Clip domain. There are 22−24 residues in the type 2 domain and 15−17 residues in type 1 domain in the CLIP-B and CLIP-C subfamilies, respectively (Kanost and Jiang, 2015).

### Spatiotemporal Expression Analysis of *Ls*CLIP3

*CLIPs* show developmental- and tissue-specific expression profiling in insects. In *P. puparum*, three *CLIPs* were highly expressed in the embryo, eight in larvae, three in pupae, and two in the adult stage (Yang et al., 2017). In this study, *Ls*CLIP3 was highly expressed in late larvae, pupae, and early adult stages, showing a potential role in metamorphosis. Similarly, the CLIP (*ApSnake*) was highly expressed in the late larval stage of *A. pernyi* (Wang et al., 2018). Homologous *CLIPs* (*snake4* and *snake11*) were highly expressed in fifth instar larvae of *Nilaparvata lugens* (Bao et al., 2014). Similar expression was also observed in *P. puparum*, in which the *Clip-SP16* was highly expressed in larvae and pupae but expressed at low levels in older mated adults (Yang et al., 2017). In *B. mori*, *CLIP-SP95* was highly expressed in larvae, but specifically in the molting process and wandering stage of pupation (Liu et al., 2017). In most insects, newly emerged adults will darken in association with the melanization process. Therefore, the high expression of *Ls*CLIP3 in newly emerged adults may be linked with the melanization pathway in *L. serricorne*. Melanization is the most rapid immune response in insects (Tang, 2009). During melanization, phenoloxidase catalyzes production of quinones to cross-link neighbor molecules and form melanin at the pathogen infection site or the injury site (Cerenius and Soderhall, 2004). We found that *Ls*CLIP3 was highly expressed in the early adult, but expressed at much lower levels in the late adult, indicating a role in cuticle melanization immediately after emergence. Melanization of the insect cuticle is also associated with the prophenoloxidase system that is regulated by hormones (Hiruma and Riddiford, 2009).

CLIPs are predominantly expressed and mainly function in insect hemolymph (Kanost and Jiang, 2015). High expression of *CLIP* in *A. pernyi* occurs in larval hemocytes (Wang et al., 2018). In *O. nipae*, three *CLIPs* were characterized and had high expression in hemocytes (Zhang et al., 2017). *CLIPs* are widely expressed in other insect tissues. *Clip-SP10* in *P. puparum* showed a higher expression level in the venom gland than other tissues analyzed, suggesting that it plays crucial roles in parasitism (Yang et al., 2017). In *B. mori*, *CLIP-SP95* was mainly expressed in cuticle, but with low expression in hemocytes (Liu et al., 2017). Similarly, we characterized high expression of two *L. serricorne CLIP-Cs* in cuticle (Chen et al., 2019). *Ls*CLIP3 is highly expressed in the integument and fat body, which are important immune-related organs during molting and metamorphosis.

### Function of *Ls*CLIP3 Involved in Molting Process

Stage-specific gene expression in insects indicates stage- or tissue-specific roles for the gene product. Larval-specific expression of *CLIP* occurs in *P. puparum* and *N. lugens* (Bao et al., 2014; Yang et al., 2017). Tissue-specific expression of *CLIP-SP95* has been demonstrated in the integument of molting stages in *B. mori* and was functionally validated in the molting process (Liu et al., 2017). The timing of gene expression in *B. mori* integument was correlated with ecdysteroid titer (Liu et al., 2017). In the present study, high expression of *Ls*CLIP3 in *L. serricorne* larvae can be induced by 20E, especially at 8 h post-treatment. This indicates that 20E-related regulation of *Ls*CLIP3 occurs during molting. In *B. mori*, the increased expression of *CLIP-SP95* has a time-dependent manner (Liu et al., 2017), which was also observed in this study, indicating a role in molting. The RNAi assay validated the role of *Ls*CLIP3 in the larval molting process. Larval–pupal molting was disrupted, and most of the test larvae died following target gene suppression. Expression of the ecdysone synthesis and signaling pathways were both inhibited in *L. serricorne*. A similar induction by 20E was investigated in chitin deacetylases (Yang et al., 2018). The phenotype of the RNAi-treated individual appears related to chitin degradation. Treated larvae were unable to molt, were malformed, and resulted in high mortality. The chitin degrading and synthesis pathway-related genes (chitin synthase) were affected. The disordered expression led to high mortality and failure to molt. This effect was also reported in *B. dorsalis*, where 20E induced the gene expressions of *CHS1* and chitinase (Yang et al., 2013a, b). Interestingly, the expression of *Ls*UAP2 was significantly increased when *Ls*CLIP3 was silenced in *L. serricorne*. In *T. castaneum*, knockdown of *Tc*UAP2 prevented larval development or led to pupal paralysis, but not affected the structural integrity of cuticle. No loss of chitin staining was observed in the peritrophic matrix of *Tc*UAP2-depleted larvae (Arakane et al., 2011). In *Locusta migratoria*, injection of *Lm*UAP2 dsRNA did not reveal any morphological abnormalities during the molting process (Liu et al., 2013). These results indicated that UAP2 may have additional biological functions besides chitin synthesis, presumably in the glycosylation of secondary metabolites or proteins (Arakane et al., 2011; Liu et al., 2013). In this study, we proposed that the increased expression of *Ls*UAP2 might be functionally linked with other metabolic pathway. However, the mechanism of this interaction is unclear and requires further investigation.

### Function of *Ls*CLIP3 Involved in Innate Immunity

The integument and fat body are immune-related organs where SPs are synthesized and function. We found that *Ls*CLIP3 was highly expressed in integument and fat body while its expression in hemolymph was not evaluated. After microbial challenge, a series of AMPs were produced in the fat body and secreted into the hemolymph. Two signaling pathways, Toll and Imd, have been implicated in the activation of downstream AMPs production (Imler et al., 2004). In insects, CLIPs in hemolymph are involved in the production of AMPs by regulating the Toll pathway (Kanost and Jiang, 2015), but their substrates are not known. In *D. melanogaster*, the Spätzle-processing enzyme (SPE) is involved in the proteolytic cascade that activates the Toll pathway. This SPE may be the bridge ligament (Roh et al., 2009). Induction of the AMP production has been demonstrated in insects. The Toll and Imd pathways activate AMP gene expression and biosynthesis. The Toll pathway is mainly induced by Gram-positive bacteria and also fungi, resulting in AMP gene expression (Imler et al., 2004; Hillyer, 2016). In *D. melanogaster*, three serine proteases (modular serine protease, grass, and SPE) are responsible for the activation of the Toll pathway that induces AMP expression (Veillard et al., 2016). An *in vivo* RNAi assay indicated that *CLIP-Spirit* is essential for SPE activation (Kambris et al., 2006). In this study, *Ls*CLIP3 expression was induced by both Gram-positive and Gram-negative bacterial PGNs, indicating a role for both Toll and Imd pathways in the host immune response. Biochemical analyses indicate a three-step proteolytic cascade of CLIPs interacting in the sensing of Gram-positive bacterial peptidoglycan (Kim et al., 2008). This induction was also observed in *A. pernyi* (Zhang et al., 2017; Wang et al., 2018), *B. mori* (Zhao et al., 2010), and *A. gambiae* (Povelones et al., 2013). In *L. serricorne*, the mortalities were increased in *Ls*CLIP3 RNAi larvae upon exposed to Gram-negative *E. coli* or Gram-positive *S. aureus*. This indicates its vital roles in innate defense responses. In *P. pernyi*, injection of the exogenous recombinant CLIP-Snake induced AMP expression (Wang et al., 2018). In contrast, suppression of *Ls*CLIP3 led to a decreased expression of four AMPs in *L. serricorne*. Therefore, our study suggest that *Ls*CLIP3 may perform antibacterial functions in *L. serricorne* by actively regulating AMP expression.

Although many CLIPs have been identified and characterized in insects, the functions of most *CLIPs* are unknown (Kanost and Jiang, 2015). In *Apis cerana cerana*, a *CLIP* gene (*AccSP1*) is found to play crucial roles in the defense of abiotic stresses and resistance to pathogens (Gao et al., 2019). In *B. mori*, only three *CLIP* genes (*CLIP1*, *CLIP2*, and *SP95*) are documented to be involved in innate immune and integument remodeling during molting (Zhao et al., 2010; Liu et al., 2017). In *Drosophila*, 18 out of 42 *CLIPs* have been functionally related to the immune Toll pathway, melanization pathway, dorsal−ventral patterning, and wing disk development (Veillard et al., 2016). The identification of the components of the Toll pathway out of the membrane and their order has been determined, while the direct protease interactions have not been confirmed *in vitro*. It is likely that additional proteins activated by CLIP proteases involved in the cascade remain uncharacterized. The current model is incomplete, although this pathway was predicted in reviews (Kanost and Jiang, 2015; Hillyer, 2016; Veillard et al., 2016).

## Data Availability Statement

The datasets generated for this study can be found in the LsCLIP3, GenBank accession number MK015721.

## Author Contributions

W-JY, K-KX, and CL conceived and designed the experiments, and wrote the manuscript. W-JY, C-XC, and YY performed the experiments. W-JY, C-XC, and K-KX analyzed the data. All authors gave final approval for the publication.

## Conflict of Interest

The authors declare that the research was conducted in the absence of any commercial or financial relationships that could be construed as a potential conflict of interest.

## Footnotes

1 https://www.ncbi.nlm.nih.gov/orffinder

2 https://blast.ncbi.nlm.nih.gov/Blast.cgi

3 http://www.expasy.ch/

4 http://smart.emblheidelberg.de/

5 http://www.cbs.dtu.dk/services/SignalP/
