# Supplementary material for: Clip-Domain Serine Protease Gene (LsCLIP3) Is Essential for Larval–Pupal Molting and Immunity in Lasioderma serricorne
Source: Front Physiol. 2020 Jan 31;10:1631. doi: 10.3389/fphys.2019.01631 (PMC7005593; doi:10.3389/fphys.2019.01631)
Supplement: Supplementary file 1 [file Data_Sheet_1.pdf]

Supplementary Material

Supplementary Tables

Supplementary Table 1. Primers used in this study.

Application of primers	Gene name	Forward primer (5′-3′)	Reverse primer (5′-3′)
ORF confirmation	*LsCLIP3*	TTATAACGAAGATGACCCACAC	CTTCCACCTCCAAACCCTTCAA
qPCR analysis	*LsCLIP3*	CCGAAAACATGGAAACTCGT	GTTCTTGTCGTCCGGATTGT
	LsCYP302a1	CACCAAAACTGCCCTTGTTT	TTTCGAAATCTTCCGGTCTG
	LsCYP306a1	GAGGAAATCGTTGGAGTGTGT	CCGTCTCTTCAATGCAACCA
	LsCYP314a1	CGACTTCTGCAACCTCATCA	CCACAAAGGCAGACCGTAAT
	LsCYP315a1	TCAGGCGTATCATGAATGGA	AGCCAGATGCTTCAATTGCT
	LsUSP	ATCACCACGCGACAACAATA	GGAACTTCAACAGCCAGAGC
	LsE74	TCCAACACCAGCACCAAGTA	TGTACGAAAGCTCGTCGTTG
	LsE75	TTCCAATTCGAGGTCTTTGG	TTCAGAGGGCAAGCAAGAGT
	LsKr-h1	CGACTTCAGCAACAACTCCA	ATCGGTGGGACGTAAGTCAG
	LsHR38	TACGACGTCGGTGGATGTTA	ATGCAACCAATCTCCGAAAG
	LsFTZ-F1	ATCGGGCGTGGTATATCGTT	CTCCGTAACGCCCATAAACG
	LsLys1	GGAACTGCGTTACGGATGTG	ACGATCTGCTTGCACTGTTG
	LsLys2	CGATTTCCGCGGAGTTTCAT	CTGTCGTCCATATTCGCACC

	LsCo1	GAGGTCCAGACTACTCGCAG	CTGAACTCCAACCCTCCAGT
	LsDef1	CGCTTTCGCTATGGTGTCTT	TGGCAGCACACAAAGAATCA
	LsDef2	TACCCAAGCTCTTCCTGTGG	GGCAAGACAATGGAGAGCAC
	LsAtt	GCAGGTCCACTTACAACAGG	AGTCCTCCGCCGTAATTAGG
	LsTRE1	CCTACGGACCAAGACCTGAA	TTTGTTGGTTGCGTTGGTAA
	LsTRE2	CGAGCTCACATCTCCAGTGA	GAGGACCTCGTATCCAGCAG
	LsUAP1	GGCAACACTGATCCATCCTT	GGCACTCTGCACGATTATGA
	LsUAP2	GCCACACAGCATTAGCAGAA	CCCAGTTTCCAGCAATCAGT
	LsCHS1	AAAGAAGAATCTGGTTCTCT	ATATAGTGCTCTCGTCACCA
	LsNAG1	CTCAAACGGACAGACTGCAA	AGTTCTGAGCGGCAAACAGT
	LsNAG2	CCCAACGACAGAGGTGTTTT	CCTCTCGCGATCAGTCTTTC
	LsCDA1	GCTTTCTTGTTTTGGGTGGA	CGAGGTCAATTTGCAGGAAT
	LsCHT5	TGAGCAGCAAGTTCCGTATG	AAGGTGAACGGCTACCATTG
	Ls18s	GTTGATCACGTCGCAAGCTA	AGGTTTCCCTCTGGCTTGTT
dsRNA synthesis	*LsCLIP3*	TAATACGACTCACTATAGGGGAAGTT GTTTGTTGCCCGGAAT	TAATACGACTCACTATAGGGCATC CTTGCTCTGTACGTTCCT
	GFP	TAATACGACTCACTATAGGGCAGTTCT TGTTGAATTAGATG	TAATACGACTCACTATAGGGAATG TTACCATCTTCTTTAA

Supplementary Table 2. Details of CLIP protein sequences used for phylogenetic analysis.

Insect species	Gene name	Accession number
Bombyx mori	*BmCLIP1*	NP_001036832
	BmCLIP2	NP_001036844
	BmCLIP6	XP_012545524
	BmCLIP7	XP_ 004926962
	BmCLIP8	XP_012548716
	BmCLIP11	NP_001037053
	BmCLIP12	XP_004927620
	BmCLIP14	XP_004925813
	BmCLIP15	XP_004931455
	BmCLIP16	XP_004930740
	BmCLIP17	NP_001040462
	BmSP95	XP_012549295
Drosophila melanogaster	*DmCG1299*	AAF47847
	DmCG6639	AAF53614
	DmCG7432	AAF55692
	DmCG8586	AAF59059
	DmCG9737	AAF57029
	DmCG15002	AAF47850
	DmCG17572	AAG22440
	DmCG31728	AAF53273
	DmMP1	AAF52151
	DmSP7	AAF54143
	DmSnake	AAF54897
	DmSpirit	AAF46392
	DmHayan	AAF48845
	DmPersephone	AAF48846
Anopheles gambiae	*AgCLIPA3*	EAA03300

	AgCLIPA4	XP_ 552464
	AgCLIPA5	XP_320729
	AgCLIPA7	XP_320723
	AgCLIPA8	XP_311445
	AgCLIPA10	XP_308802
	AgCLIPB8	XP_312743
	AgCLIPB9	XP_003436374
	AgCLIPB10	XP_ 312744
	AgCLIPC1	XP_552698
	AgCLIPC2	XP_313588
	AgCLIPC3	XP_313589
	AgCLIPD2	XP_317284
	AgCLIPD3	XP_321698
	AgCLIPD4	XP_312102
	AgSP14D	AAB62929. 1
Apis mellifera	*AmSPH41*	XP_006563756
	AmSPH55	XP_001120817
	AmSP1	XP_ 006570267
	AmSP2	XM_006570203
	AmSP7	XP_625051
	AmSP10	XP_001120043
	AmSP14	XP_001121032
	AmSP21	XP_006567247
	AmSP33	XP_ 006559393
Manduca sexta	*MsHP6*	AAV91004. 1
	MsHP21	AAV91019. 1
	MsSPH1	AAM69352.2
	MsSPH2	AAM69353.1
	MsPAP1	AAX18636. 1
	MsPAP2	AAL76085. 1

	MsPAP3	AAO74570. 1
	MsSP140	XP_030022322.1
	MsSP142	XP_030022368.1
	MsSP143	XP_030022342.1
Lasioderma serricorne	*LsCLIP1*	MK015723
	LsCLIP2	MK015722
	LsCLIP3	MK015721
Leptinotarsa decemlineata	*LdSPeaster-like*	XP_023025567.1
Agrilus planipennis	*ApCLIP2*	XP_018332507.1
Tribolium castaneum	*TcSPP136*	EFA07558. 1
Tenebrio molitor	*TmPPAF*	CAC12696. 1
	Tm41kDa	BAG14261.1
